# Outcomes of melphalan 140 mg/m^2^ followed by autologous stem cell transplantation in multiple myeloma patients with co‐morbidities: Single‐centre experience

**DOI:** 10.1002/jha2.977

**Published:** 2024-08-19

**Authors:** Dario Melotti, Samir Asher, Ethan Troy‐Barnes, George Nesr, William Wilson, Marquita Camilleri, Rakesh Popat, Ke Xu, Neil Rabin, Jonathan Sive, Xenofon Papanikolaou, Lydia Lee, Annabel McMillan, Kwee Yong, Chara Kyriakou

**Affiliations:** ^1^ Haematology University College Hospital London UK; ^2^ Department of Haematology University College London Hospitals NHS Foundation Trust London UK; ^3^ Clinical Haematology Imperial College Healthcare NHS Trust London UK; ^4^ CRUK and UCL Cancer Trials Centre University College London London UK; ^5^ Haematology Bristol Haematology and Oncology Centre Bristol UK; ^6^ Haematology University College London Hospitals NHS Foundation Trust London UK

**Keywords:** haematology, multiple myeloma, transplantation

## Abstract

High‐dose melphalan followed by stem cell rescue is the standard consolidative therapy for transplant‐eligible patients with multiple myeloma (MM) in the United Kingdom. A melphalan dose of 200 mg/m^2^ (Mel200) is considered the “gold standard” for autologous stem cell transplant (ASCT) conditioning for fit patients ≤70 years old; however, with a peak diagnosis incidence at 80–89 years old in the UK dose adjustments will be inevitable to limit toxicities. In this single‐centre UK‐based retrospective analysis, data was collected from patients with plasma cell dyscrasias who underwent a first reduced‐intensity, Mel140, ASCT from 2006 to 2019, a total of 81 patients. We found that the procedure was overall safe with seven (9%) of patients requiring ITU admission and a single transplant‐related death within the initial autograft admission. The progression‐free survival (PFS) and overall survival were comparable with those previously reported in the literature with median PFS for our cohort of 31 months. Univariate analysis of our data showed an inferior PFS for patients aged ≥70 years. In conclusion, although this is a retrospective analysis, it demonstrates that dose‐reduced melphalan conditioning is safe and effective in patients deemed unfit for standard‐intensity conditioning.

1

High‐dose melphalan followed by stem cell rescue is the standard consolidative therapy for transplant‐eligible patients with multiple myeloma (MM) in the United Kingdom [1]. A melphalan dose of 200 mg/m^2^ (Mel200) is considered the “gold standard” for autologous stem cell transplant (ASCT) conditioning for fit patients ≤70 years old [2]; however, with a peak diagnosis incidence at 805–89 years old in the UK [3] and the consequent increase in comorbidities with age [4], dose adjustments will be inevitable to limit toxicities.

A number of studies [5, 6, 7, 8, 9, 10, 11] compared outcomes using a melphalan dose of 140 mg/m^2^ (Mel140) to Mel200, with contradicting results for progression‐free (PFS) and overall survival (OS) as well as treatment‐emergent toxicities. The differences in mobilization regimens and post‐transplant maintenance therapy amongst these studies are worth highlighting as these may have confounded the reported results.

Herein, we retrospectively analysed the outcomes of patients who received Mel140 conditioning in our institution between 2006 and 2020.

In this single‐centre UK‐based retrospective analysis, data was collected from patients with plasma cell dyscrasias (MM; n = 77, AL amyloidosis; *n* = 1, POEMS syndrome; *n* = 3) who underwent a first reduced‐intensity, Mel140 and ASCT from 2006 to 2019. Patients were selected for Mel140 based on age, renal impairment or other comorbidities considered too high‐risk for a Mel200 ASCT. All patients received Mel140 on day −1 followed by haemopoietic stem cell infusion on day 0. Granulocyte‐colony stimulating factor was started on D+6, according to local protocol. The response was recorded according to the International Myeloma Working Group criteria. Time to neutrophil engraftment was defined as time to neutrophil counts recover to > 0.5 × 10^9^/L. High‐risk (HR) cytogenetics were defined as the presence of t4;14, t14;16 and/or tp53/17p del.

The primary objective was to assess the modified Mel140‐related toxicity in patients considered too frail to receive Mel200. The secondary objectives were OS and PFS.

Median PFS, and OS and corresponding 95% confidence intervals (95% CIs) were calculated using Kaplan–Meier methods. PFS and OS were defined as the time from ASCT to the date of disease progression and death from any cause, respectively. Patients had disease restaging 100 days post‐ASCT as per local guidelines and thereafter routinely in MM clinics. Data was available for 81 patients (50 males and 31 females). The median age was 62 years (range 41–75, interquartile range [IQR] 1). Note that, 62/75 patients (83%) with available data were known to have comorbidities. Note that, 22/81 (27%) had chronic kidney disease (CKD) stage IV (glomerular filtration rate [GFR] 15–29) or V (GFR < 15) and 5/40 (12.5%) had a Karnofsky Performance Scale (PS) of ≤80 (median PS: 90). International Staging System (ISS) score was II‐III in 46/59 patients (78%) and 10/31 patients (32%) had HR cytogenetics. Note that, 57/81 patients (70%) received a single prior line of induction MM treatment (range 0–3) before proceeding to transplant. Prior to ASCT, induction treatment included proteasome inhibitor‐containing regimens in 61/81 (75%) and immunomodulatory drugs‐containing regimens in 46/81 (57%) of patients.

Of the 81 patients who received Mel140 ASCT, 7 (9%) required ITU transfer during their ASCT admission (sepsis [*n* = 4], type 1 respiratory failure [*n* = 2] and cardiac complications [*n* = 1]). There was one transplant‐related mortality (TRM) during the initial transplant admission (day +26) having developed neutropenic sepsis and an ischaemic Cerebrovascular Accident secondary to an Middle Cerebral Artery thrombus.

The median time to neutrophil engraftment was 11 days (range 9–15, IQR 1).

The median PFS was 2.3 years (95% CI 1.8–3.8) and the median OS was 6.2 years (95% CI 5.6–N/A) (Figure 1). Early relapse, defined as biochemical or radiological progression < 18 months from D0, was observed in 17 (21%) patients.

**FIGURE 1:**
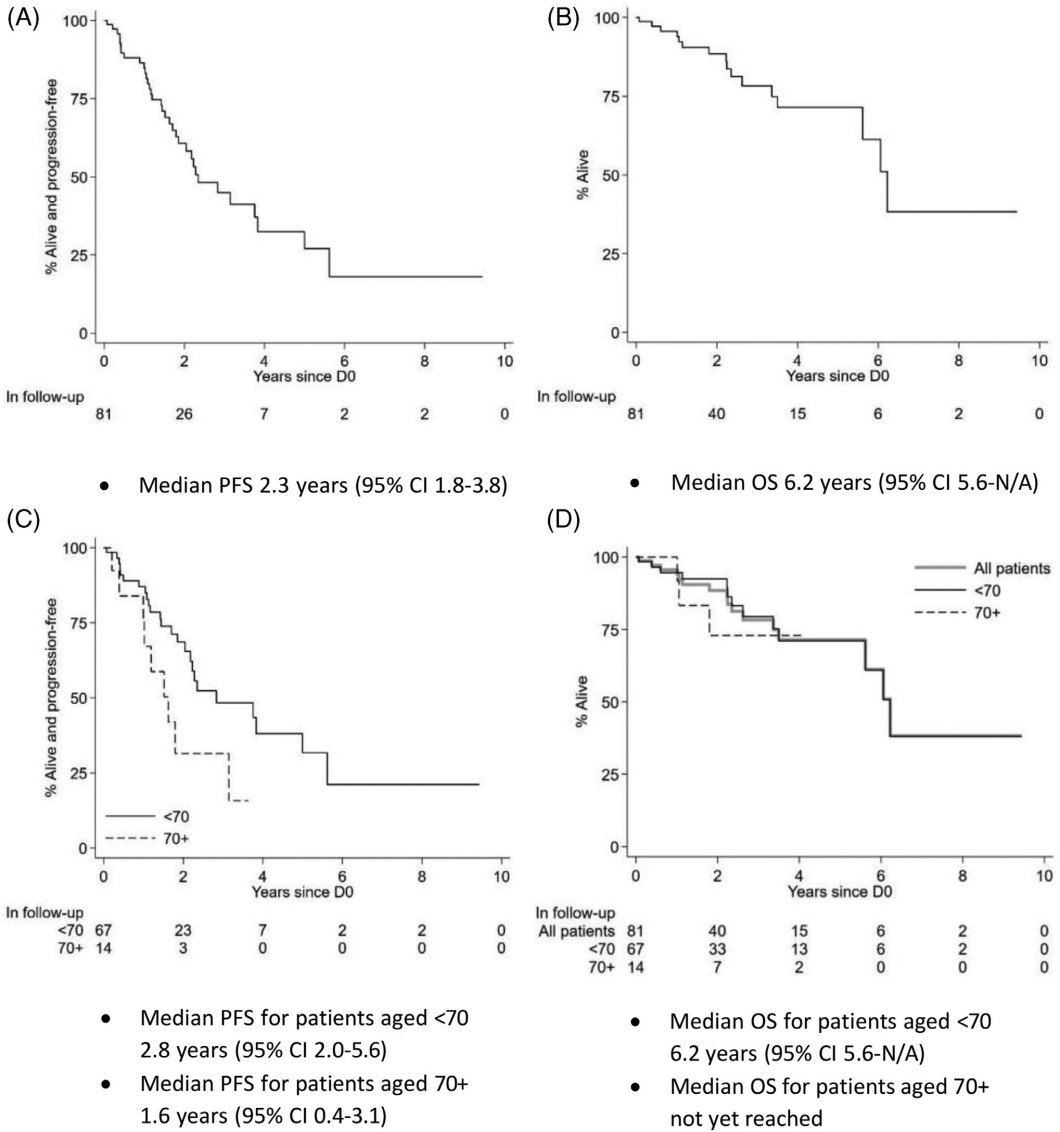
(A) Progression‐free survival (PSF), (B) overall survival (OS), (C) PFS by age, and (D) OS by age.

Univariate analysis was performed for age at ASCT ≥65 and ≥70 years, presence of comorbidities, left ventricular ejection fraction < 50%, pre‐existing dialysis‐dependence, CKD IV, HR cytogenetics, ISS/revised ISS II‐III, Karnofski PS, HCT‐CI > 2 and > 0 (Table 1). Among the above, PFS was inferior for patients aged ≥70 versus < 70 years (median PFS 2.8 years vs 1.6 years, HR = 2.23, *p* = 0.045). There was no significant impact of age on OS. No other variables reached statistical significance.

**TABLE 1:** Univariate analysis of risk factors on progression‐free survival and overall survival.

		PFS			OS		
Risk factor	*N* = 81	Had event	HR (95% CI)	*p*‐Value	Had event	HR (95% CI)	*p*‐Value
**Age at ASCT ≥65**							
Yes	35 (43.2%)	16	1.52 (0.77–2.99)	0.231	6	1.05 (0.37–2.94)	0.931
No	46 (56.8%)	18			10		
**Age at ASCT ≥70**							
Yes	14 (17.3%)	9	2.23 (1.02–4.87)	0.045	3	1.28 (0.35–4.67)	0.704
No	67 (82.7%)	25			13		
**Co‐morbidities**							
Yes	62 (82.7%)	30	1.54 (0.54–4.43)	0.421	15	2.19 (0.28–16.89)	0.451
No	13 (17.3%)	4			1		
Unknown	6						
**LVEF < 50%**							
Yes	5 (6.5%)	2	1.01 (0.23–4.40)	0.991	2	3.77 (0.80–17.82)	0.094
No	72 (93.5%)	30			12		
Unknown	4						
**RRT**							
Yes	7 (8.6%)	2	0.82 (0.19–3.44)	0.784	1	0.78 (0.10–5.99)	0.810
No	74 (91.4%)	32			15		
**Stage 4 CKD (eGFR < 30 mL/min)**							
Yes	22 (27.2%)	5	0.54 (0.21–1.40)	0.203	3	0.61 (0.17–2.21)	0.452
No	59 (72.8%)	29			13		
**Poor risk cyto (t4;14, t14;16 and/or tp53/17p del)**							
Yes	10 (32.3%)	7	1.81 (0.68–4.81)	0.234	5	2.93 (0.79–10.96)	0.109
No	21 (67.7%)	10			4		
Unknown	50						
**Poor risk ISS (II or III)**							
Yes	46 (78.0%)	22	0.41 (0.17–1.00)	0.050	8	0.27 (0.08–0.90)	0.033
No	13 (22.0%)	7			5		
Unknown	22						
**Poor risk R‐ISS (II or III)**							
Yes	16 (80.0%)	7	0.55 (0.13–2.34)	0.417	4	0.63 (0.11–3.50)	0.601
No	4 (20.0%)	3			2		
Unknown	61						
**Karnofsky PS (< 80)**							
Yes	5 (12.5%)	2	0.54 (0.12–2.37)	0.415	0	N/A	N/A
No	35 (87.5%)	17			8		
Unknown	41						
**IMWG VGPR or better**							
Yes	57 (70.4%)	21	0.75 (0.37–1.53)	0.429	10	0.91 (0.33–2.52)	0.853
No	24 (29.6%)	13			6		
**HCT‐CI > 2**							
Yes	8 (9.9%)	2	0.51 (0.12–2.14)	0.355	1	0.64 (0.08–4.96)	0.670
No	73 (90.1%)	32			15		
**HCT‐CI > 0**							
Yes	50 (61.7%)	21	1.08 (0.54–2.17)	0.823	11	1.56 (0.54–4.50)	0.414
No	31 (38.3%)	13			5		

Abbreviation: HCT‐CI, Hematopoietic Cell Transplantation Comorbidity Index; IMWG VGPR, Very Good Partial Response according to International Myeloma Working Group criterias; LVEF, Left ventricular ejection fraction; RRT, Renal replacement therapy.

Although the practice of dose‐reducing melphalan conditioning for ASCT in patients with comorbidities is well established, there is limited published data available regarding outcomes for this approach. This study recorded our experience with a cohort of 81 patients, which is large for a single‐centre study when compared to available data. Our data confirms this remains a safe approach for patients with co‐morbidities with only one recorded TRM.

The PFS and OS were comparable with those previously reported in the literature. The median PFS for our cohort was 31 months. Katragadda et al. [10] reported a median PFS of 31.2 months in patients receiving a Mel140 autograft. A larger multi‐centre study coordinated by the EBMT Chronic Malignancies Working Party that included 245 patients with MM receiving Mel140ASCT reported a median PFS of 29 months, while median OS was not reached [11].

Univariate analysis of our data, with the limitation of the low subgroup numbers, showed an inferior PFS for patients aged ≥70 years. Both of the aforementioned studies reported that age > 65 did not affect either OS or PFS (a finding replicated in our study) but did not specifically report on the ≥70 age group. However, this difference in PFS did not translate into a difference in OS between the age groups. It is possible this was due to the shorter follow‐up and a smaller number of patients in the > 70 age group.

In conclusion, although this is a retrospective analysis, it demonstrates that dose‐reduced melphalan conditioning is safe and effective in patients deemed unfit for standard‐intensity conditioning. Co‐morbidities including impaired cardiac left ventricle function, CKD, pre‐existing dialysis dependence and HR genetics did not affect patient outcomes in this limited dataset. The PFS and OS were comparable to existing data, and although patients over 70 showed a reduced PFS, their OS was unaffected. While this should be interpreted within the limitations mentioned above, it suggests that patients in this age group should still be considered for ASCT if otherwise fit.

## CONFLICT OF INTEREST STATEMENT

The authors declare no conflict ofinterest. The authors did not receive support from an organisation for the submitted work.

## FUNDING INFORMATION

The authors received no specific funding for this work.

## ETHICS STATEMENT

This project was a departmental service development audit approved by local departmental governance. As this was a retrospective study reporting on outcomes of standard of care treatment, no additional ethical approval was required.

## PATIENT CONSENT STATEMENT

The authors have confirmed patient consent statement is not needed for this submission.

## CLINICAL TRIAL REGISTRATION

The authors have confirmed clinical trial registration is not needed for this submission.

## Data Availability

The data that support the findings of this study are available from the corresponding author upon reasonable request.
